# Supplementary material for: Comparative genomics provides new insights into the diversity, physiology, and sexuality of the only industrially exploited tremellomycete: Phaffia rhodozyma
Source: BMC Genomics. 2016 Nov 9;17:901. doi: 10.1186/s12864-016-3244-7 (PMC5103461; doi:10.1186/s12864-016-3244-7)
Supplement: Additional file 6: — List of orphan genes with links to PFAM (related to Additional file 1: Table S1). (ZIP 1428 kb) [file 12864_2016_3244_MOESM6_ESM.zip › BLAST_HTML_FTR/G00141_P.html]

BLAST Search Results


```
BLASTP 2.2.27+


Reference:
Stephen F. Altschul, Thomas L. Madden, Alejandro A. Schäffer,
Jinghui Zhang, Zheng Zhang, Webb Miller, and David J. Lipman (1997),
"Gapped BLAST and PSI-BLAST: a new generation of protein database
search programs", Nucleic Acids Res. 25:3389-3402.


Reference for
composition-based statistics:
Alejandro A. Schäffer, L. Aravind, Thomas L. Madden, Sergei
Shavirin, John L. Spouge, Yuri I. Wolf, Eugene V. Koonin, and
Stephen F. Altschul (2001), "Improving the accuracy of PSI-BLAST
protein database searches with composition-based statistics and
other refinements", Nucleic Acids Res. 29:2994-3005.


Database: nr
           71,551,133 sequences; 26,053,659,533 total letters


Query= G00141_P

Length=364
                                                                      Score     E
Sequences producing significant alignments:                          (Bits)  Value

emb|CED83987.1|  hypothetical protein [Xanthophyllomyces dendrorh...   714    0.0  
ref|WP_004057192.1|  glycosyl transferase [Haloferax mediterranei...  38.9    7.6  


 >emb|CED83987.1| hypothetical protein [Xanthophyllomyces dendrorhous]
Length=363

 Score =  714 bits (1843),  Expect = 0.0, Method: Compositional matrix adjust.
 Identities = 363/363 (100%), Positives = 363/363 (100%), Gaps = 0/363 (0%)

Query  1    MYKSNHALARSDPTSAPPFYPDSYLRVIPSPSLSSPGPSVRYVRNLTKSPSTNTLPSSSL  60
            MYKSNHALARSDPTSAPPFYPDSYLRVIPSPSLSSPGPSVRYVRNLTKSPSTNTLPSSSL
Sbjct  1    MYKSNHALARSDPTSAPPFYPDSYLRVIPSPSLSSPGPSVRYVRNLTKSPSTNTLPSSSL  60

Query  61   PIRSPSNLRVPAYSIIHPISSTLQRQKHVSRVPSRATHLQHIRDTLPAVMHGQTNRVTEP  120
            PIRSPSNLRVPAYSIIHPISSTLQRQKHVSRVPSRATHLQHIRDTLPAVMHGQTNRVTEP
Sbjct  61   PIRSPSNLRVPAYSIIHPISSTLQRQKHVSRVPSRATHLQHIRDTLPAVMHGQTNRVTEP  120

Query  121  LPWTVDLGDDSEFSHSVETKQRTVRQASDPMPLTSTGSLVPPRPSRNVDMVFGSRAGRPV  180
            LPWTVDLGDDSEFSHSVETKQRTVRQASDPMPLTSTGSLVPPRPSRNVDMVFGSRAGRPV
Sbjct  121  LPWTVDLGDDSEFSHSVETKQRTVRQASDPMPLTSTGSLVPPRPSRNVDMVFGSRAGRPV  180

Query  181  RRVSSVPVTLDDRYIFSVNPNAVPPCSPPPKYSIVPAPSVNGSSTSYLLSPRSRTLPYMP  240
            RRVSSVPVTLDDRYIFSVNPNAVPPCSPPPKYSIVPAPSVNGSSTSYLLSPRSRTLPYMP
Sbjct  181  RRVSSVPVTLDDRYIFSVNPNAVPPCSPPPKYSIVPAPSVNGSSTSYLLSPRSRTLPYMP  240

Query  241  MERSDSSCSEDGSYSSVSPLPITPLYSVEDNSPRLRLSSSTTSISSTSICSFSPRDEDDK  300
            MERSDSSCSEDGSYSSVSPLPITPLYSVEDNSPRLRLSSSTTSISSTSICSFSPRDEDDK
Sbjct  241  MERSDSSCSEDGSYSSVSPLPITPLYSVEDNSPRLRLSSSTTSISSTSICSFSPRDEDDK  300

Query  301  DLFDLLPAEQDDGLNRPIETFLHDIDELSMSVWPDEAGHIILLPPVPLKSHKRAHQHTSD  360
            DLFDLLPAEQDDGLNRPIETFLHDIDELSMSVWPDEAGHIILLPPVPLKSHKRAHQHTSD
Sbjct  301  DLFDLLPAEQDDGLNRPIETFLHDIDELSMSVWPDEAGHIILLPPVPLKSHKRAHQHTSD  360

Query  361  VLS  363
            VLS
Sbjct  361  VLS  363


>ref|WP_004057192.1| glycosyl transferase [Haloferax mediterranei]
 gb|AFK19058.1| glycosyl transferase group 1 [Haloferax mediterranei ATCC 33500]
 gb|EMA04047.1| group 1 glycosyl transferase [Haloferax mediterranei ATCC 33500]
 gb|AHZ21583.1| glycosyl transferase [Haloferax mediterranei ATCC 33500]
Length=354

 Score = 38.9 bits (89),  Expect = 7.6, Method: Compositional matrix adjust.
 Identities = 38/139 (27%), Positives = 67/139 (48%), Gaps = 13/139 (9%)

Query  70   VPAYSIIHPISSTLQRQKHVSRVPSRATHLQHIRDTLPAVMHGQTNR-----VTEPLPWT  124
            +P  S++H ++S L+      R   RA   +++      V + +T +     VT+P P  
Sbjct  88   IPVVSVVHHLNS-LENLGTFRRYIRRAIETRYLHSVDAFVFNSETTKETVSAVTDPDPSV  146

Query  125  VDL--GDD-SEFSHSVETKQRTVRQASDPMPLTSTGSLVPPRPSRNVD-MVFGSRAGRPV  180
            V    GD  SE+   ++ ++   R A  P+ L S G+L    P +N+D ++ G    R  
Sbjct  147  VAYPAGDRFSEYGTPLDDEEIRARVAEGPLRLISVGNL---EPRKNIDGLLRGLARVRDD  203

Query  181  RRVSSVPVTLDDRYIFSVN  199
              V+ V  T+DD Y+ S++
Sbjct  204  WEVTVVGATVDDEYVVSLH  222


Lambda      K        H        a         alpha
   0.314    0.130    0.387    0.792     4.96 

Gapped
Lambda      K        H        a         alpha    sigma
   0.267   0.0410    0.140     1.90     42.6     43.6 

Effective search space used: 3217712111204


  Database: nr
    Posted date:  Sep 23, 2015 12:05 AM
  Number of letters in database: 26,053,659,533
  Number of sequences in database:  71,551,133


Matrix: BLOSUM62
Gap Penalties: Existence: 11, Extension: 1
Neighboring words threshold: 11
Window for multiple hits: 40
```
